# F185. BRAIN STRUCTURAL PATTERNS DIFFERENTIATE EARLY- AND LATE-ONSET CANNABIS USE IN PSYCHOTIC PATIENTS: PRELIMINARY RESULTS FROM THE PRONIA STUDY

**DOI:** 10.1093/schbul/sby017.716

**Published:** 2018-04-01

**Authors:** Nora Penzel, Peter Falkai, Nikolaos Koutsouleris, Joseph Kambeitz

**Affiliations:** Ludwig Maximilian University

## Abstract

**Background:**

Cannabis use is considered to be one of the most important environmental risk factors for developing psychosis. Previous research indicates that consumption which is initiated during adolescence is associated with a higher risk of adverse effects. It has been proposed that in this period cannabis use may be more harmful due to the disruptive impact on the endocannabinoid system which is critically involved in brain development. Multiple studies indicate structural brain alterations in patients with schizophrenia as well as in cannabis users. However, the effect of cannabis use on brain development in patients with psychosis is currently only poorly understood. Thus, in the current analysis we employed a multivariate approach to investigate the hypothesis that early cannabis use might be associated with marked alterations in brain structure that are distinct from alterations in late-users.

**Methods:**

n=39 patients with recent onset psychosis (ROP) and cannabis abuse of the PRONIA sample took part in a structural MRI (sMRI) scan and were clinically assessed with respect to their cannabis use characteristics and psychotic symptoms using the positive and negative symptoms scale (PANSS). Patients were grouped into early-users (n=21, onset before age of 18) and late-users (n=18, onset after age of 18). Multivariate pattern classification was performed on the basis of sMRI data to differentiate early- and late-users.

**Results:**

Early- and late-users did not differ with respect to age, gender or amount of current cannabis use. Early-users showed significantly higher PANSS scores compared to late-users (p < 0.05). Structural MRI allowed the differentiation between early- and late-users with 72 % (81 % of the early-users, 61 % of the late-users).

**Discussion:**

The current results indicate a distinction between psychotic patients with cannabis abuse who started to consume before the age of 18 and those who did later in life. The groups could be distinguished by means both of their clinical data, i.e., more severe psychotic symptoms in the early-users, and of their neuroanatomical data. These findings are in line with former literature, indicating that cannabis use during the period of adolescence is associated with persistent and more severe negative outcomes than use which is initiated in the adulthood. However, due to the relatively low sample size, these results serve only as preliminary results and need further investigation.

